# BMI1 activates P-glycoprotein via transcription repression of *miR-3682-3p* and enhances chemoresistance of bladder cancer cell

**DOI:** 10.18632/aging.203277

**Published:** 2021-07-16

**Authors:** Ming-Kun Chen, Jun-Hao Zhou, Peng Wang, Yun-lin Ye, Yang Liu, Jia-Wei Zhou, Zi-Jian Chen, Jian-Kun Yang, De-Ying Liao, Zhi-Jian Liang, Xiao Xie, Qi-Zhao Zhou, Kang-Yi Xue, Wen-Bin Guo, Ming Xia, Ji-Ming Bao, Cheng Yang, Hai-Feng Duan, Hong-Yi Wang, Zhi-Peng Huang, Zi-Ke Qin, Cun-Dong Liu

**Affiliations:** 1 Department of Urology, The Third Affiliated Hospital of Southern Medical University, Guangzhou 510630, China; 2 Department of Pathology, Cancer Center, Sun Yat-Sen University, Guangzhou 510060, China

**Keywords:** BMI1, chemoresistance, P-glycoprotein, miR-3682-3p, bladder cancer

## Abstract

Chemoresistance is the most significant reason for the failure of cancer treatment following radical cystectomy. The response rate to the first-line chemotherapy of cisplatin and gemcitabine does not exceed 50%. In our previous research, elevated BMI1 (B-cell specific Moloney murine leukemia virus integration region 1) expression in bladder cancer conferred poor survival and was associated with chemoresistance. Herein, via analysis of The Cancer Genome Atlas database and validation of clinical samples, BMI1 was elevated in patients with bladder cancer resistant to cisplatin and gemcitabine, which conferred tumor relapse and progression. Consistently, BMI1 was markedly increased in the established cisplatin- and gemcitabine-resistant T24 cells (T24/DDP&GEM). Functionally, BMI1 overexpression dramatically promoted drug efflux, enhanced viability and decreased apoptosis of bladder cancer cells upon treatment with cisplatin or gemcitabine, whereas BMI1 downregulation reversed this effect. Mechanically, upon interaction with p53, BMI1 was recruited on the promoter of *miR-3682-3p* gene concomitant with an increase in the mono-ubiquitination of histone H2A lysine 119, leading to transcription repression of *miR-3682-3p* gene followed by derepression of *ABCB1* (ATP binding cassette subfamily B member 1) gene. Moreover, suppression of P-glycoprotein by miR-3682-3p mimics or its inhibitor XR-9576, could significantly reverse chemoresistance of T24/DDP&GEM cells. These results provided a novel insight into a portion of the mechanism underlying BMI1-mediated chemoresistance in bladder cancer.

## INTRODUCTION

Bladder cancer is the 2^nd^ most common tumor in urinary system. There are 549,000 new cases of bladder cancer in the world, and 200,000 people die from this disease each year [1]. About 30% of patients are initially diagnosed with muscle-invasive bladder cancer (MIBC) [2]. Radical cystectomy complemented with chemotherapy is the main treatment option for MIBC, particularly for high-risk patients. The adjuvant chemotherapy can effectively repress the growth and prevent the recurrence of tumor [3, 4]. Cisplatin (DDP) and gemcitabine (GEM) chemotherapy (GC chemotherapy) is used as the first-line treatment for patients with advanced MIBC. However, the response rate to platinum-based regimens does not exceed 50% [3]. Multi-drug resistance (MDR) is the main cause of the failure in cancer chemotherapy and is essential for cancer metastasis and recurrence [5–8]. The potential mechanisms of MDR currently consists of the ABC transporter family, miRNA regulation, cancer stem cell initiation and so on [9]. Intracellular drug transport, as the primary mechanism of MDR in cancer cells, is mediated by MDR related genes, like P-glycoprotein (P-GP) and MDR-associated protein 1 (MRP1) [10, 11]. However, the molecular mechanism underpinning the modulation of MDR related genes is still largely unclear.

MiRNAs are non-coding ~22 nucleotide RNAs that regulate genes expression at post-transcriptional level. As another important mechanism of MDR, miRNAs take part in MDR regulation by modulating target genes [9]. For instance, miR-19a/b is upregulated in gastric cancer and regulated MDR by inhibiting PTEN [12]. Ectopic miR-153 in colorectal cancer mediated drug resistance by targeting forkhead box O3a (FOXO3a) [13]. Hence, the role of miRNA regulation in MDR, especially in MDR related genes, deserves to be further explored.

B-cell specific Moloney murine leukemia virus integration region 1 (BMI1) is the main component of the polycomb group complex 1 (PRC1), which functions as an important epigenetic inhibitor of various regulatory genes associated with chemoresistance, embryogenesis, self-renewal, senescence and so on [14–18]. BMI1 is an oncogene and its aberrant expression is associated with numerous cancers and resistance to certain chemotherapies, which confers poor prognosis [19–21]. Many studies have demonstrated that BMI1 can stimulate cancer initiation [22], cell transformation [23] as well as induce epithelial–mesenchymal transition [24–26]. Inhibiting BMI1 can make cancer cells sensitive to chemotherapy through induction of AKT-mediated apoptosis pathway [27–29]. In bladder cancer, BMI1 reduction inhibits cell proliferation, migration, invasion [30, 31], stemness properties and tumorigenicity [32, 33]. In our previous research, elevated BMI1 expression in bladder cancer was correlated with poor overall survival [34]. Further biological analyses of The Cancer Genome Atlas (TCGA) database revealed *BMI1* was increased in bladder cancer patients resistant to chemotherapy, which confers tumor relapse and progression. The oncogenic role of BMI1 in chemoresistance of bladder cancer deserves to be further characterized.

This research investigated the oncogenic roles of BMI1 in GC-chemoresistant bladder cancer and potential functions of miRNAs in BMI1-mediated activation of P-GP in chemoresistant bladder cancer.

## MATERIALS AND METHODS

### Cell culture and establishment of resistant cell line

T24 and BIU-87 cells were purchased from American Type Culture Collection (ATCC) and were cultured base on the instructions. T24 cells were grown in McCoy's 5A modified medium (Gibco, Grand Island, NY, USA, Cat. No. 16600082) and BIU-87 cells were grown in RPMI-1640 (Gibco, Cat. No. 22400071) with of 10% fetal bovine serum added (FBS, Biofluids, Camarillo, CA, USA). All bladder cancer cells were cultured in the atmosphere of 5% carbon dioxide at 37° C.

To isolate cisplatin- and gemcitabine-resistant T24 cells, 2 × 10^7^ cells were seeded in medium supplemented with cisplatin (DDP) at 0.05 μg/ml and incubated for 24h. The residual living cells were expanded over 3 days without DDP followed by treatment with gemcitabine (GEM) at 0.2 μg/ml for 24h. The residual viable cells were expanded over 3 days in normal medium. A second and third round of selection was performed in a similar manner with increasing concentration of DDP and GEM. Finally, the resistant cells could be stably cultured in medium with DDP at 0.5 μg/ml or GEM at 2.5 μg/ml.

### Clinical tissues and patient information

240 paraffin sections of bladder cancer tissues and 8 fresh bladder cancer specimens were collected at Sun Yat-Sen University Cancer Center from 2000 to 2010. The clinical data of these specimens are shown in Table 1. Prior informed consents were obtained from patients and the approvals by the Institutional Research Ethics Committee were gained.

**Table 1:** Correlation between BMI1 expression and clinicopathological characteristics of 240 bladder cancer specimens.

**Characteristics**		**BMI1**		***P*-value**
		**Higher expression**	**Lower expression**	
**BMI expression**	-	118	122	-
**Gender**	male	80	87	0.55
	female	38	35	
**Age (years)**	≤ 65	55	59	0.76
	> 65	63	63	
**No. of tumors**	single	90	92	0.87
	multiple	28	30	
**Stage**	T2	5	12	< 0.001
	T3	45	67	
	T4	68	33	
**Grade**	low	41	80	< 0.001
	high	77	42	
**Lymph node**	positive	61	43	0.005
	negative	40	70	
	not available	17	19	

### Immunohistochemistry

Immunohistochemical analysis (IHC) was carried out in 240 human bladder cancer tissues using an anti-BMI1 (Abcam, Cat. No. ab126783) and anti-P-GP (Abcam, Cat. No. ab170904) as described previously [35, 36]. The immunostaining degree of paraffin sections was evaluated and averaged by two independent researchers, who were not informed of histopathological characteristics and patients’ information. The positive degree of staining was assessed: 0 (no positive tumor cells (PTCs)), 1 (<10% PTCs), 2 (10-50% PTCs), and 3 (>50% PTCs). The intensive degree of staining was estimated: 0 (no staining), 1 (weak, light yellow), 2 (moderate, yellowish brown), and 3 (strong, brown). The staining index (SI) was calculated: staining index = staining intensity × proportion of positive tumors staining. According to the heterogeneity measurement of relapse-free survival rate by log-rank test statistics, the cut-off value was selected to define the high or low expression of BMI1. An optimal cut-off value was determined. The SI ≥ 6 was defined as high-expressing tumours, and the SI < 6 was defined as low-expressing tumours.

### Cell proliferation/cytotoxicity test

Cells were treated with/without DDP or GEM at indicated concentration for 48 hours. At the end of drug exposure, 10 μl Cell Counting Kit-8 (BS350A, Biosharp, Hefei, Anhui, China) solutions were supplemented to each well including 100 μl medium. After 1.5-hour incubation absorbance was measured at 450 nm.

### Cell cycle assay

Wash cells twice by ice-cold phosphate buffer saline and fix them overnight with cold 70% ethanol. And then the cells were mixed with 100 μg/ml propidium iodide (PI) and incubated in dark for 30 minutes. Upon addition with 400 μl phosphate buffer saline, cells were analyzed by flow cytometry for cycle detection.

### Dye efflux test

The cells in six-well plates were washed twice by phosphate buffer saline. Then cells were incubated with 2 μg/ml rhodamine 123 (Sigma-Aldrich, Cat. No. R8004) in the dark for 30 minutes. After that, cells were visualized by inverted fluorescence microscope every 10 min or analyzed by flow cytometry for detection of fluorescence levels of rhodamine 123. The experiments were carried out in triplicate. The experimental results were consistent and considered to be statistically significant.

### Annexin V apoptosis assay

The apoptosis detecting kits (KeyGEN Biotech, Nanjing, China) were applied to detect tumor cell apoptosis. In short, cells were implanted in a six-well plate, cultured for 24 h and supplemented with at 5 μg/ml or GEM at 25 μg/ml. The cells were added with 5 μl Annexin V-FITC, and then treated with 5 μl PI reagents. Upon addition of 400 μl phosphate buffer saline, the cells were examined by flow cytometry to detect apoptosis cells. Annexin V-FITC-positive but PI-negative cells were regarded as apoptosis cells.

### Real-time quantitative reverse transcription (qRT-PCR)

Extract RNA from cells or bladder cancer tissues using TRIzol reagent (Invitrogen). Transcribe reversely RNA with a miRNA 1^st^-Strand Synthesis Kit (TaKaRa, Cat.NO.638313) for miRNA detection. The qRT-PCR for miRNA was carried out on an Mx3005P thermal cycler using a PCR kit (TaKaRa, Cat. NO. RR820A). Sample intensities were normalized to RNU6B snRNA abundance. Data were analyzed using Applied Mx3005P Software and using the relative cycles to threshold method. The primer used for detecting miR-3682-3p expression was listed as follows: TGATGATACAGGTGGAGGTAGGT.

### Western blotting (WB) analysis

WB was carried out base on the standard protocol, as mentioned earlier [37]. The primary antibodies were applied as followed: anti-BMI1 (Abcam, Cat. No. ab126783), anti-P-GP (Abcam, Cat. No. ab170904) and anti-p53 (Abcam, Cat. No. ab1101). Glyceraldehyde-3-phosphate dehydrogenase (GAPDH, Proteintech, Cat. No. 60004-1-Ig) was used as a loading control.

### Plasmids, retrovirus infection, and transduction

*BMI1* gene was amplified by PCR from cDNA and cloned into pSin-EF2 vectors. Three BMI1-targeting shRNA sequences (RNAi#1: AGAACAGATTGGATCGGAA; and RNAi#2: AGACCACTACTGAATATAA; RNAi#3: TACATTTATACCTGGAGAA) were cloned into SUPER.retro.puro vectors (OligoEngine, USA) to construct the respective pSUPER.retro.BMI1-RNAi(s). T24 or BIU-87 cells were seeded in the P100 plate, and then transducted with 10 μg plasmids. Cell lines stably expressing BMI1 or BMI1 shRNA were constructed through retrovirus infection of HEK293T cells and selected using 0.5 mg/ml puromycin.

### CRISPR/cas9 generation of BMI1−/− cells

Lentiviral CRISPR–Cas9 vectors that mediated *BMI1* gene editing were purchased from Beyotime Biotechnology (Beyotime, Shanghai, China). The targeted sequence of sgRNA was GACAATACTTGCTGGTCTCC. After 48h lentiviral transfection, cells were screened using puromycin. To screen for clones with BMI1 gene disruption, total genomic DNA was extracted and genomic PCR of *BMI1* gene was performed with primers: forward: 5'-CCACCTGATGTGTGTGCTTTG-3'; reversed: 5'-TTCAGTAGTGGTCTGGTCTTGT-3'. PCR products were analysed on 1% agarose gel supplemented with ethidium-bromide and immunoblots were performed to confirm BMI1 depletion.

### miRNA microarray assay analysis

miRNA expression profiles of two different cell samples (T24/DDP&GEM vs. T24/DDP&GEM-sgBMI1 cells) were established by SHBIO Technology Corporation (Shanghai, China). The procedures were carried out base on manufacturer's recommendations.

### Prediction of miRNAs targeting *ABCB1* gene

MiRWalk 2.0, a comprehensive database of predicted and validated miRNA-target interactions, was used to predict the binding sites by microRNAs in 3’-UTR of *ABCB1* gene [38].

### Luciferase reporter test

293T cells were implanted in 96-well plate for 24h. *ABCB1* 3’-UTR-luciferase plasmid and renilla plasmid (Promega) were transducted into cells with lipofectamine 3000 (Invitrogen). Then the luciferase and renilla signals were detected with Dual Luciferasel Reporter Test Kit (Promega) base on the protocols.

### Co-immunoprecipitation (co-IP) assay

The indicated cells were lysed, and then immunoprecipitated by anti-BMI1 (Abcam, Cat. No. ab126783) and anti-p53 (Abcam, Cat. No. ab1101) antibodies. Then the immune complexes were detected by anti-BMI1 (Abcam, Cat. No. ab126783) and p53 (Abcam, Cat. No. ab1101) antibodies, and visualized with an ECL analysis system.

### Chromatin immunoprecipitation (ChIP)

ChIP test was carried out with an EZ ChlP Kit (Abcam, Cambridge, MA, USA, Cat. No. ab500) base on the protocol. In short, 5×10^6^ cells were cross-linked with 1% formaldehyde for 15 minutes. Cross-linked chromatin was sonicated and then incubated using anti-IgG, anti-p53 (Abcam, Cat. No. ab1101), anti-BMI1 (Abcam, No. ab126783) or anti-H2AK119ub1 (CST, Cat. No. 8240). The immunoprecipitated DNA was purified and quantified by RT-PCR to detect the binding level of p53, which was standardized to 0.5% input. The primer used for ChlP test was listed below: *miR-3682-3p* promoter:

5'-GGTTTACAGATAAGACTGGGAATG-3',

5'-CTTTCTGCCCATTTCCAC-3'.

### Statistical analysis

The statistical methods contained *t* test, Fisher’s exact test, χ^2^ test and log-rank test. Univariate and multivariate analyses were used with a Cox regression model. Statistical analysis was carried out using SPSS 11.0 software. The data represented mean ± SD; *P* ≤ 0.05 was considered to be statistically significant.

## RESULTS

### Ectopic BMI1 in bladder cancer tissues from patients resistant to chemotherapy correlated with poor prognosis

**Figure 1:**
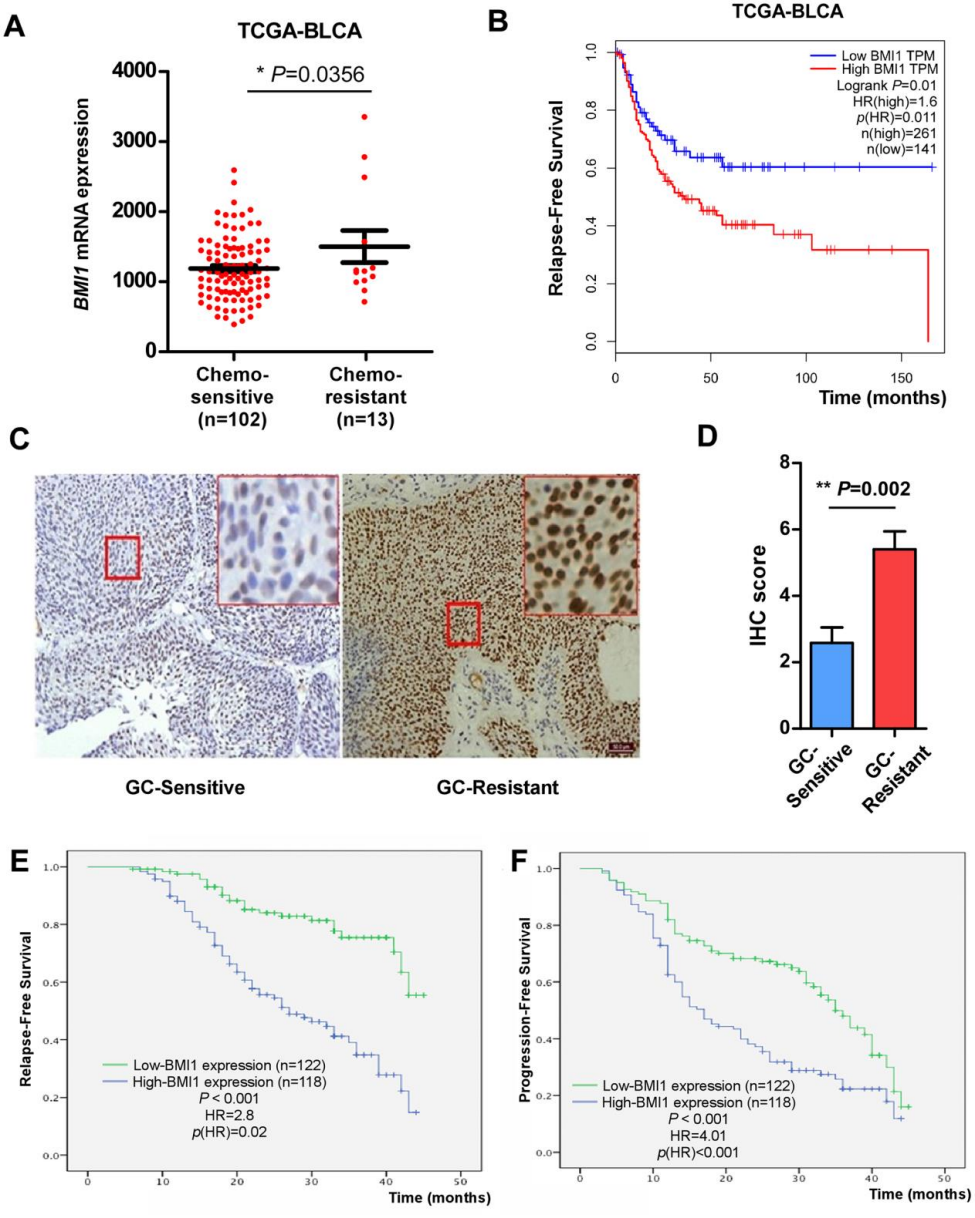
**Elevated BMI1 in GC-chemoresistant bladder cancer conferred poor prognosis.** (**A**) *BMI1* mRNA expression in bladder cancer tissues of patients partial response to chemotherapy versus patients complete response to chemotherapy from TCGA-BLCA database. (**B**) Relapse-free survival of patients in TCGA-BLCA dataset with low versus high levels of *BMI1* mRNA. (**C**) IHC analysis of BMI1 protein expression in bladder cancer tissues of patients resistant to GC chemotherapy and that sensitive to GC chemotherapy, magnification, ×200 & ×400. (**D**) Statistical quantification of the IHC score of BMI1 staining in bladder cancer specimens from patients resistant versus sensitive to GC chemotherapy. (**E**) Relapse-free survival of patients with bladder cancer with low versus high BMI1 expression. (**F**) Progression-free survival of patients with bladder cancer with low versus high BMI1 expression. *P < 0.05. GC: Cisplatin and Gemcitabine; TCGA: The Cancer Genome Atlas; BLCA: Bladder Urothelial Carcinoma; IHC: Immunohistochemistry; TPM: Transcripts Per Million.

In our previous research, elevated *BMI1* expression was related to poor overall survival in bladder cancer [34]. For further investigating the regulation role of *BMI1* in human bladder cancer, we firstly analysed *BMI1* levels in bladder cancer tissues from TCGA-bladder urothelial carcinoma (BLCA) database. According to the available clinical information from TCGA-BLCA dataset, 102 patients with complete response to chemotherapy and 13 patients with partial response to chemotherapy were included for analysis. Here, patients with complete response to chemotherapy were defined as chemo-sensitive patients, while patients with partial response to chemotherapy were defined as chemo-resistance. *BMI1* expression was significantly elevated in 13 patients resistant to chemotherapy compared with that in 102 patients sensitive to chemotherapy (Figure 1A), suggesting the association of *BMI1* with chemoresistance. In particular, patients with high *BMI1* levels had a worse relapse-free survival rate than patients with low *BMI1* levels (Figure 1B). These data suggested that ectopic *BMI1* may be an indicator of chemoresistance and poor prognosis of bladder cancer.

In order to verify the above analysis, we examined BMI1 expression in 240 archived, paraffin-embedded bladder cancer specimens using IHC staining. Consistently, the level of BMI1 in tumor tissues from patients resistant to GC chemotherapy was significantly higher than that sensitive to GC chemotherapy (Figure 1C). Quantitative IHC score analysis further corroborated the elevated BMI1 expression in bladder cancer tissues from patients resistant to GC chemotherapy (Figure 1D).

The association of BMI1 level and the clinicopathologic characteristics of bladder cancer were further evaluated among 240 bladder cancer specimens. 118 (49.17%) had high BMI1 expression, while 122 (50.83%) had low BMI1 expression (Table 1). The statistical analyses indicated that upregulated BMI1 expression was related to higher clinical stage, grade and lymph node (LN) metastasis (*P* < 0.01), but not with gender, age or multiplicity (Table 1). BMI1 expressions in bladder cancer tissues were inversely related to relapse-free survival (*P* < 0.001, Figure 1E) and progression-free survival (*P* < 0.001, Figure 1F). Furthermore, the expression level of BMI1 was an independent prognosis factor for bladder cancer (*P* < 0.05) similar to clinical grade, stage, multiplicity and LN metastasis (Tables 2, 3). Taken together, BMI1 upregulation in bladder cancer involves in chemoresistance and is related to poor prognosis for bladder cancer.

**Table 2:** Univariate and multivariate analyses of tumor-specific mortality in patients with bladder cancer by cox-regression analysis.

**Characteristics**	**Univariate**			**Multivariate**	
	**HR (95%CI)**	***P*-value**		**HR (95%CI)**	***P*-value**
**Gender:** male, female	0.85 (0.62, 1.11)	0.361		-	-
**Age (years):** ≤ 65, > 65	0.81 (0/62, 1.02)	0.141		-	-
**No. of tumors:** single, multiple	3.17 (1.76, 5.37)	0.021		2.57 (1.62, 3.13)	0.022
**Stage:** T2, T3, T4	3.56 (1.63, 4.93)	0.011		3.13 (1.29, 5.61)	0.003
**Grade:** low, high	2.77 (1.32, 4.05)	0.033		2.99 (1.73, 5.03)	0.035
**Lymph node:** negative, positive	3.79 (2.32, 5.29)	0.001		3.58 (1.93, 6.02)	0.003
**BMI1 expression**	2.81 (1.61, 4.02)	0.019		2.73 (1.93, 3.85)	0.013

**Table 3:** Univariate and multivariate analyses of tumor progression in patients with bladder cancer by cox-regression analysis.

**Characteristics**	**Univariate**			**Multivariate**	
	**HR (95%CI)**	***P*-value**		**HR (95%CI)**	***P*-value**
**Gender:** male, female	0.94 (0.81, 1.09)	0.213		-	-
**Age (years):** ≤ 65, > 65	0.84 (0.67, 1.19)	0.421		-	-
**No. of tumors:** single, multiple	1.93 (1.51, 3.07)	0.021		1.73 (1.24, 2.39)	0.047
**Stage:** T2, T3, T4	2.85 (1.79, 4.21)	< 0.001		2.39 (1.88, 3.77)	0.003
**Grade:** low, high	1.45 (1.03, 1.93)	0.043		2.33 (1.66, 3.30)	0.041
**Lymph node:** negative, positive	3.72 (1.99, 5.35)	< 0.001		2.79 (1.53, 4.33)	0.001
**BMI1 expression**	4.01 (2.01, 7.31)	< 0.001		3.03 (1.73, 5.37)	0.005

### Inhibition of BMI1 up-regulation reversed the chemoresistance in T24/DDP&GEM cells

**Figure 2:**
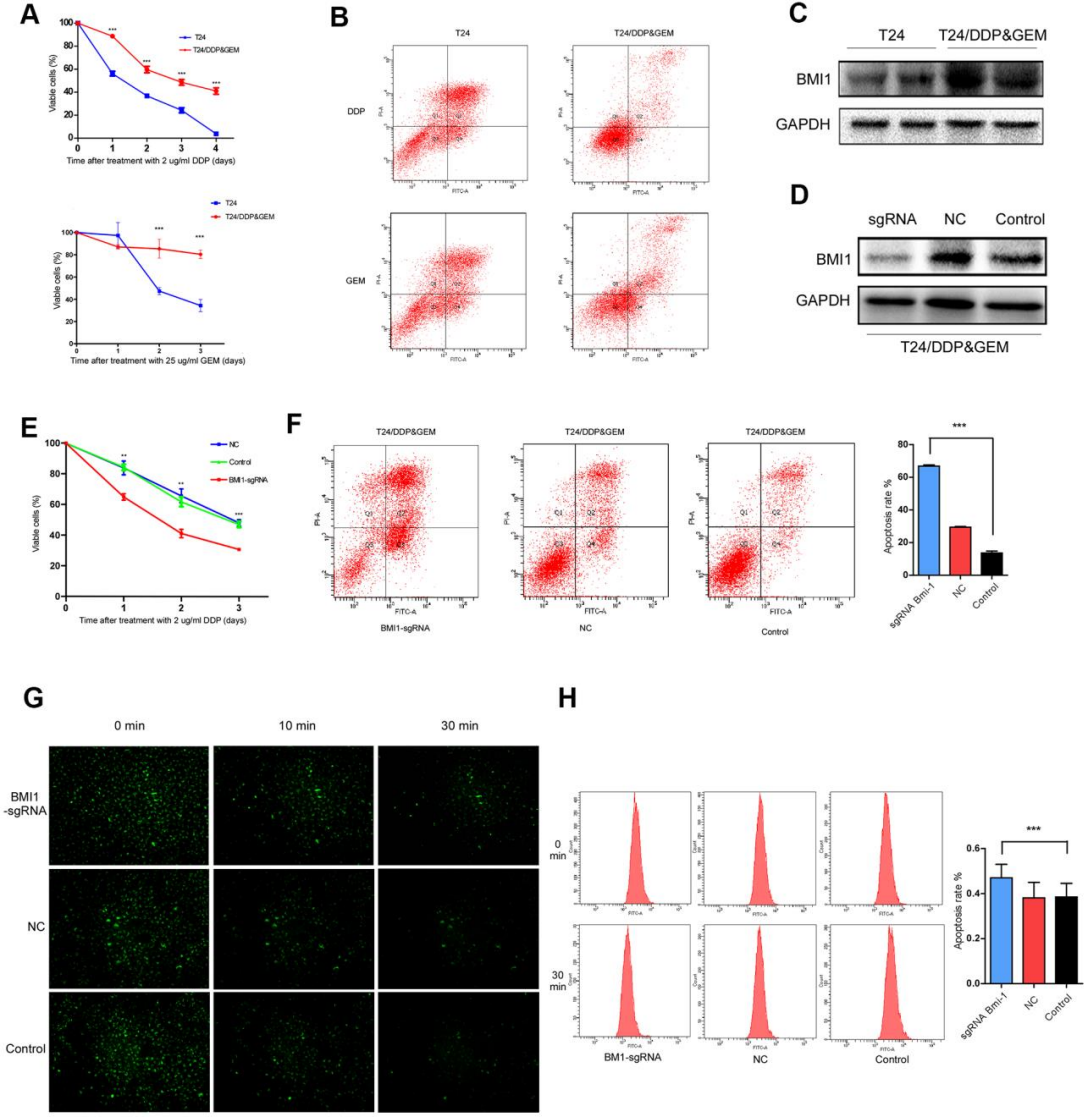
**Inhibition of BMI1 overexpression reversed the chemoresistance in GC-resistant T24 cells.** (**A**) Cell proliferation changes of T24 and T24/DDP&GEM cells assessed by cell counting kit-8 assays after treatment with 2 μg/ml DDP or 25 μg/ml GEM. (**B**) Apoptosis of T24 and T24/DDP&GEM cells detected by the Annexin V/flow cytometric apoptosis assay after treatment with 2 μg/ml DDP or 25 μg/ml GEM. (**C**) Western Blot analysis of BMI1 protein in T24 and T24/DDP&GEM cells. (**D**) Western Blot detection of BMI1 protein in T24/DDP&GEM cells upon BMI1 knockout using CRISPR/Cas9 system. (**E**) Cell proliferation changes of T24/DDP&GEM cells upon knockout of BMI1 were assessed by cell counting kit-8 assays after treatment with 2 μg/ml DDP. (**F**) Apoptosis of T24/DDP&GEM-sgBMI1 and T24/DDP&GEM cells detected by the Annexin V/flow cytometric apoptosis assay after treatment with 2 μg/ml DDP. (**G**) Under observation in inverted fluorescence microscope, the fluorescent intensity of dye in indicated cells after transfection with rhodamine 123. (**H**) The retention rate of rhodamine 123 dye in down-regulating BMI1 or vector control T24/DDP&GEM cells detected by flow cytometry. *P < 0.05. **P < 0.01. ***P < 0.001. GC: cisplatin and gemcitabine; DDP: cisplatin; GEM: gemcitabine.

In order to better mimic the characteristics of clinical bladder cancer resistant to GC chemotherapy, we established a bladder cancer cell lines, named T24/DDP&GEM, resistant to cisplatin and gemcitabine by alternately exposing T24 cells to cisplatin or gemcitabine. The drug resistance of T24/DDP&GEM cells was confirmed by cell proliferation and apoptosis assays upon treatment with cisplatin or gemcitabine (Figure 2A, 2B). The half maximal inhibitory concentration (IC_50_) of T24/DDP&GEM cells for cisplatin and gemcitabine was significantly increased compared with T24 cells (Table 4). Of note, BMI1 expression was significantly higher in resistant T24/DDP&GEM cells than that in T24 cells (Figure 2C), which was consistent with the elevated BMI1 expression in bladder cancer tissues of patients resistant to GC chemotherapy. These data supported the crucial role of BMI1 in GC chemoresistant bladder cancer.

**Table 4:** The half maximal inhibitory concentration (IC_50_) values and resistance index (RI) of T24/DDP&GEM and T24 cells.

**Cells**	**IC_50_ (μg/ml)**	
	**Cisplatin**	**Gemcitabine**
**T24/DDP&GEM**	4.93 ± 0.42	45.97 ± 3.35
**T24**	1.16 ± 0.24	0.73 ± 0.12
RI (T24/DDP&GEM : T24)	4.25	62.97

DDP: Cisplatin; GEM: Gemcitabine; T24/DDP&GEM cells: cisplatin- and gemcitabine-resistant T24 cells.

For further investigating the biological role of BMI1 in GC chemoresistant bladder cancer, we knock-downed BMI1 in T24/DDP&GEM cells, which was named T24/DDP&GEM-sgBMI1 cells using CRISPR/Cas9 system (Figure 2D). Intriguingly, BMI1 knockdown reversed the chemoresistance of T24/DDP&GEM cells. Depletion of BMI1 reduced viability and promoted apoptosis of T24/DDP&GEM cells when treated with cisplatin (Figure 2E, 2F). Under observation in inverted fluorescence microscope, the fluorescent intensity of rhodamine 123 dye was decreased more slowly in T24/DDP&GEM-sgBMI1 cells than that in T24/DDP&GEM cells (Figure 2G). Consistently, the retention rate of fluorescent dye was elevated in T24/DDP&GEM-sgBMI1 cells as detected by flow cytometry (Figure 2H), which suggested that BMI1 mediated chemoresistance of bladder cancer probably via promotion of drug efflux.

### BMI1 enhanced the chemoresistance in bladder cancer cells

**Figure 3:**
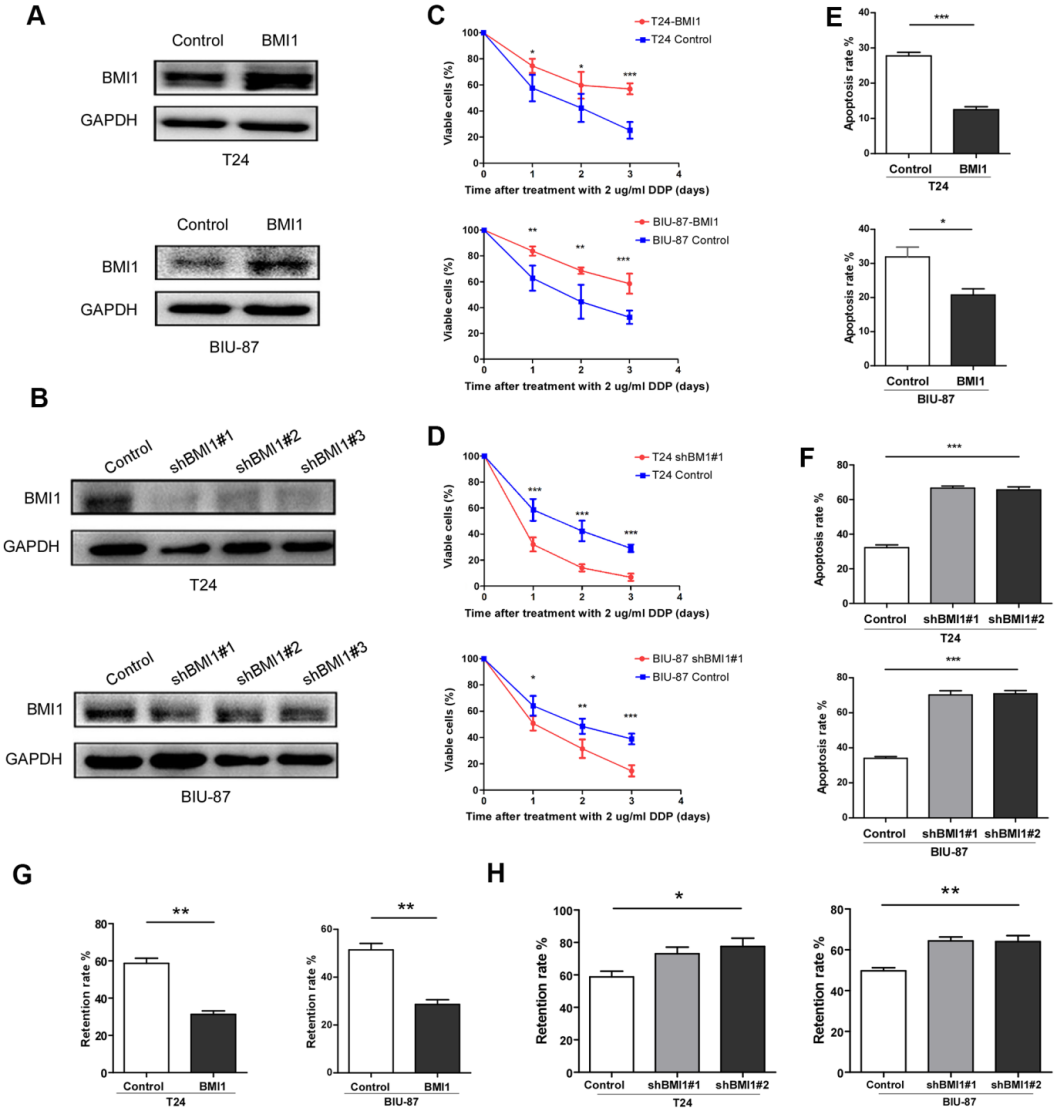
**BMI1 enhanced the chemoresistance of bladder cancer cells.** (**A**, **B**) Western blot analysis of BMI1 in the indicated BMI1-transduced, BMI1-silenced, or vector control cells. GAPDH was used as the loading control. (**C**, **D**) Cell proliferation changes of BMI1-overexpressing, BMI-silenced or vector control cells assessed by cell counting kit-8 assays after treatment with 2 μg/ml DDP. (**E**, **F**) Apoptosis of T24 and BIU-87 cells upon BMI1 up-regulation or knock-down was determined by the Annexin V/flow cytometric apoptosis assay after treatment with 2 μg/ml DDP for 72h. (**G**, **H**) The retention rate of rhodamine 123 in up-regulating BMI1, down-regulating BMI1 or vector control cells detected by flow cytometry. *P < 0.05. GAPDH: glyceraldehyde3-phosphate dehydrogenase; DDP: cisplatin.

For investigating the roles of BMI1 in bladder cancer chemoresistance, we knock-downed or overexpressed BMI1 in T24 and BIU-87 cells, respectively (Figure 3A, 3B). Upon treatment with cisplatin, BMI1-overexpressing cells presented a higher viability and lower apoptosis as assessed by the cell counting kit-8 analysis (Figure 3C) and Annexin V/flow cytometry apoptosis assay (Figure 3E), whereas BMI1-downregulating cells reduced the viability and increased the apoptosis rate (Figure 3D, 3F). These results indicated that BMI1 could enhance chemoresistance of bladder cancer cells, while BMI1 knockout could increase the sensitivity to chemotherapy. Furthermore, as detected by flow cytometry, the retention rate of fluorescent dye rhodamine 123 was significantly decreased in BMI1-overexpressing cells (Figure 3G), whereas significantly increased in BMI-silenced cells (Figure 3H). These findings supported the above-mentioned viewpoint that BMI1 increased chemoresistance probably via promotion of drug efflux, which was associated with ABC transporter family. Taking together, BMI1 knockdown reversed the chemoresistance of T24/DDP&GEM cells.

### miR-3682-3p directly suppressed *ABCB1* gene, contributing to BMI1-mediated chemoresistance in bladder cancer cells.

**Figure 4:**
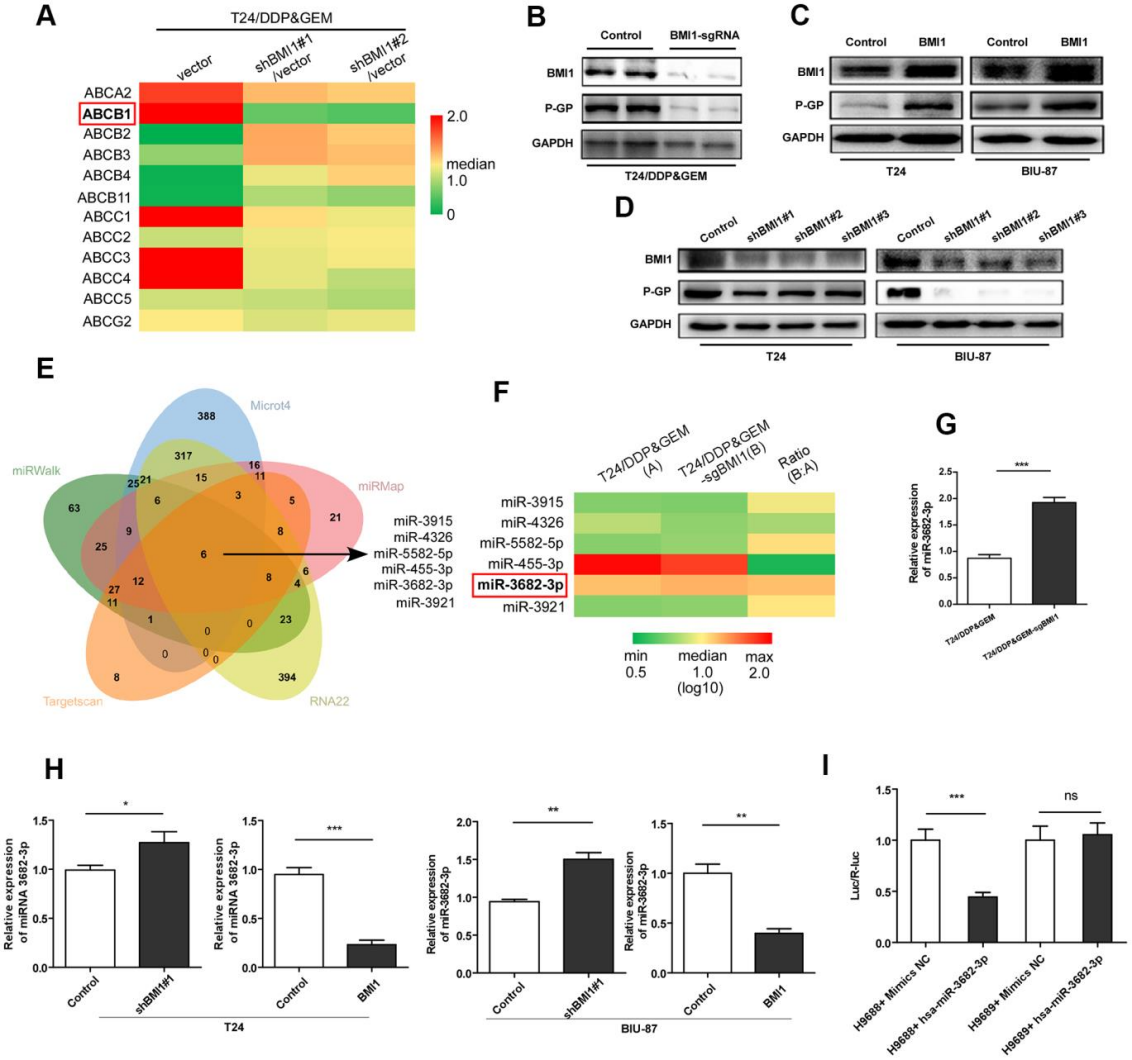
**BMI1 activated P- glycoprotein via suppression of *miR-3682-3p*.** (**A**) qRT-PCR detection of 12 human ABC-transporters, which are associated with drug transport, in T24/DDP&GEM cells upon BMI1 knockdown. (**B**) Western blot detection of BMI1 and P-GP in T24/DDP&GEM and T24/DDP&GEM-sgBMI1 cells. (**C**, **D**) Western blot analysis of BMI1 and P-GP in overexpressing, downregulating BMI1 or vector control cells (T24 and BIU-87). (**E**) A Venn diagram showing the overlap of candidate miRNAs that were predicted by miRWalk2.0 to potentially bind to the *ABCB1* 3'-UTR. (**F**) Heatmap of 6 candidate miRNAs expression in miRNA microarray assay analysis between T24/DDP&GEM and T24/DDP&GEM-sgBMI1 cells. (**G**) Quantification analysis of miR-3682-3p expression by q-RT-PCR in T24/DDP&GEM and T24/DDP&GEM-sgBMI1 cells. (**H**) Detection of miR-3682-3p expression by q-RT-PCR in overexpressing, downregulating or vector control cells (T24 and BIU-87). (**I**) Results of luciferase reporter assay in HEK293T cells with co-transfection of *ABCB1* 3'-UTR vector (H9688) or mutant control vector (H9689). *P < 0.05. **P < 0.01. ***P < 0.001. DDP: cisplatin; GEM: gemcitabine; P-GP: P-glycoprotein.

Next we explored the potential mechanism underpinning BMI1-mediated promotion of drug efflux, which was associated with ABC transporter family. This family has at least 48 members [39], 12 of which are considered to be putative drug transporters [40]. Then these 12 human ABC-transporters were examined by qRT-PCR and *ABCB1* gene level was significantly decreased in T24/DDP&GEM cells upon BMI1 knockdown (Figure 4A). Western Blot assay further confirmed the reduction of P-GP protein in T24/DDP&GEM-sgBMI1 cells (Figure 4B). Analogously, BMI1 overexpression increased P-GP protein in bladder cancer cells (Figure 4C), whereas BMI1 knockdown decreased its expression (Figure 4D).

Given the important regulatory role of miRNAs in silencing of gene expression, especially in MDR related genes [9], we explored the potential regulation of miRNAs in P-GP reduction. A comprehensive database, miRWalk 2.0, including miRWalk, RNA22, miRMap, Microt4, and TargetScan, were applied to predict miRNAs potentially inhibiting *ABCB1* gene by binding to its 3'-UTR. And then, six candidate microRNAs (miR-3915, miR-4326, miR-5582-5p, miR-455-3p, miR-3682-3p, and miR-3921) were predicted (Figure 4E). Notably, among these microRNAs, miR-3682-3p was detected by miRNA arrays to be significantly over-expressed in T24/DDP&GEM-sgBMI1 cells relative to T24/DDP&GEM cells (Figure 4F). Subsequent q-RT-PCR further confirmed the increased expression of miR-3682-3p in T24/DDP&GEM-sgBMI1 cells (Figure 4G). Consistently, in another two bladder cancer cells, T24 and BIU-87, miR-3682-3p was detected by q-RT-PCR to be highly expressed upon BMI1 knockdown, whereas miR-3682-3p was down-regulated after overexpressing BMI1 (Figure 4H). To investigate whether miR-3682-3p can specifically inhibit *ABCB1* gene, we constructed an *ABCB1* 3'-UTR plasmid (H9688) that included a 3'-UTR of *ABCB1* downstream of the luciferase gene and a mutant plasmid (H9689) that contained a mutant site in predicted binding site. Luciferase reporter assay showed that compared to the control groups, miR-3682-3p markedly inhibited luciferase activities of reporters including 3'-UTR of *ABCB1*, while miR-3682-3p had no influence on luciferase activities of reporters including the mutant 3'-UTR of *ABCB1* (Figure 4I). These data indicated that *ABCB1* was a miR-3682-3p target and miR-3682-3p was involved in BMI1-mediated chemoresistance by directly binding to *ABCB1* 3'-UTR and suppressing its expression.

### BMI1 epigenetically repressed transcription of *miR-3682-3p* upon interaction with p53

**Figure 5:**
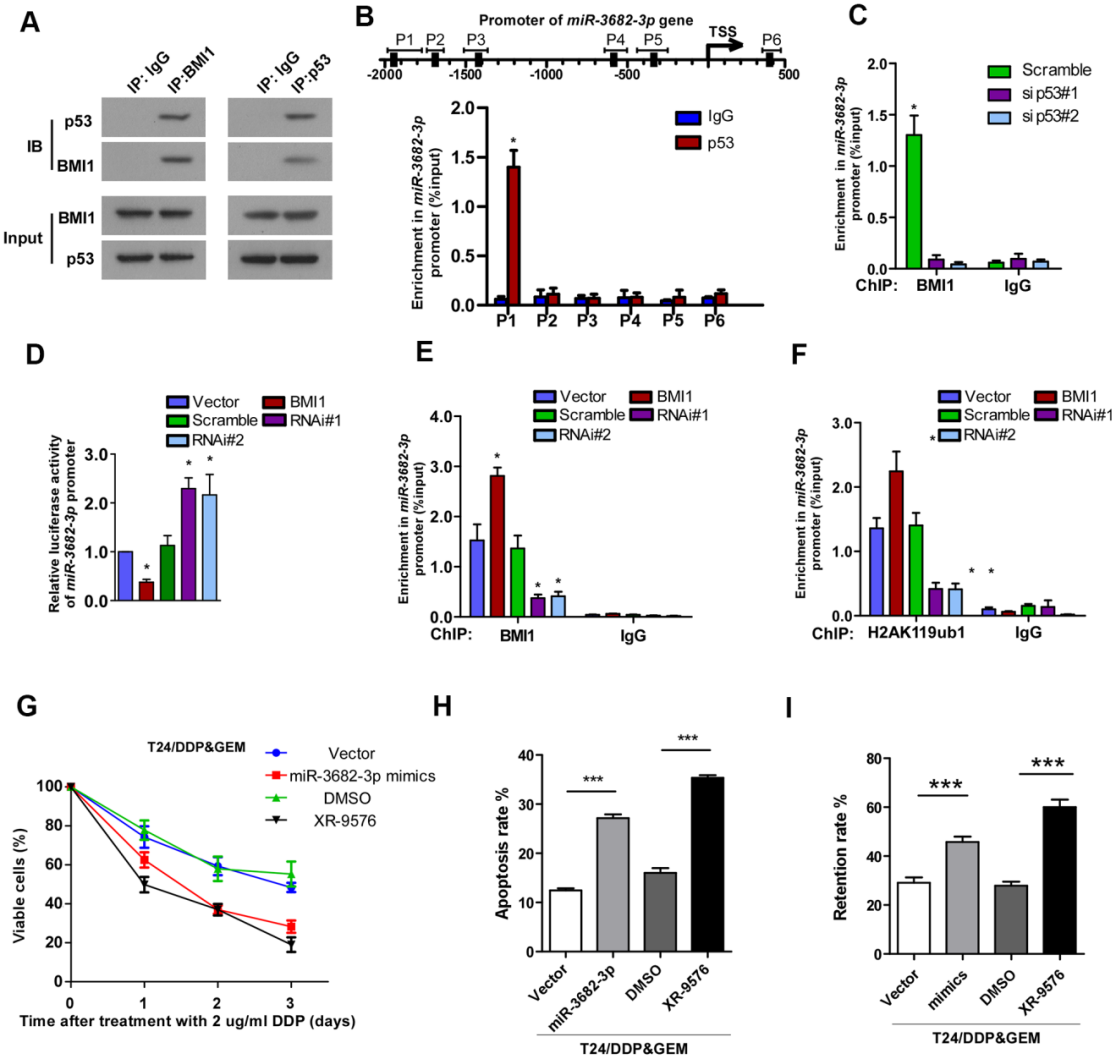
**Interacted with p53, BMI1 epigenetically repressed transcription of *miR-3682-3p*.** (**A**) Co-IP assay showing the interaction of BMI1 with p53 in T24 cells. (**B**) ChIP assay showing the nucleotide regions of *miR-3682-3p* promoter that are physically associated with p53. Upper panel: schematic illustration of predicted p53-bound sites and PCR-amplified fragments of the *miR-3682-3p* promoter; lower panel: ChIP assays were performed using p53 antibody to validate p53-bound *miR-3682-3p* promoter regions. IgG was used as a negative control. (**C**) ChIP-qPCR analysis showing enrichment of p53 at *miR-3682-3p* promoter in the indicated cells. (**D**) *miR-3682-3p* promoter luciferase reporter plasmids, Renilla pRL-TK plasmids, vector, or BMI1 were transfected into T24 cells. After 48 h, cells were subjected to a luciferase reporter assay. (**E**, **F**) ChIP-qPCR analysis of BMI1 (**E**) and H2AK119ub1 (**F**) at promoter of *miR-3682-3p* in T24 cells. (**G**) Cell proliferation changes of T24/DDP&GEM cells transfected with miR-3682-3p mimics, *ABCB1* inhibitor XR-9576, or negative control were assessed by cell counting kit-8 assays after treatment with 2 μg/ml DDP. (**H**) Apoptosis of the indicated cells detected by the Annexin V/flow cytometric apoptosis assay after treatment with 2 μg/ml DDP. (**I**) The retention rate of Rhodamine 123 in the indicated cells detected by flow cytometry. *P < 0.05. **P < 0.01. ***P < 0.001. Co-IP: co-immunoprecipitation; ChIP: chromatin immunoprecipitation; DDP: cisplatin.

Next we investigated the potential mechanism underlying BMI1-mediated reduction of miR-3682-3p. Given the crucial role of BMI1 in transcriptionally silencing genes [41], we supposed that BMI1 repressed *miR-3682-3p* transcription probably via interference with its regulators. Then we predicted the potential transcription regulators that could interact with BMI1, as well as regulate *miR-3682-3p* expression by Biological General Repository for Interactionh Datasets (BioGRID) and JASPAR database. Of these proteins, p53 aroused great interest as it have been reported to interact with BMI1 [42], and BMI1 loss results in the upregulation of p53 targets [43, 44]. Herein, co-IP assay validated the interaction of BMI1 with p53 in T24 cells (Figure 5A). According to the predicted binding sites of *miR-3682-3p* gene, ChIP assay was performed and confirmed that p53 could directly associate with the promoter of *miR-3682-3p* gene (Figure 5B). Interestingly, silencing p53 significantly decreased BMI1 occupancy on *miR-3682-3p* gene promoter (Figure 5C). Furthermore, BMI1 overexpression decreased *miR-3682-3p* promoter-driven reporter activity in T24 cells, whereas silencing BMI1 had the opposite effects (Figure 5D). These results presented evidence that BMI1 could interact with p53 and repress *miR-3682-3p* transcription.

BMI1 is a major component of PRC1, which was shown to function as a transcriptional repressor through catalyzing the mono-ubiquitination of histone H2A lysine 119 (H2AK119ub1), an epigenetic marker related to gene suppression [45–47]. ChIP showed that BMI1 occupancy on the *miR-3682-3p* promoter was increased and accompanied by increased H2AK119ub1 upon BMI1 up-regulation (Figure 5E, 5F). Consistent with these results, RNAi of BMI1 significantly decreased the occupancy of BMI1 on the promoter as well as those of H2AK119ub1 (Figure 5E, 5F). Collectively, these results suggested that BMI1, interacted with p53, could repress the transcription of *miR-3682-3p* through H2AK119 mono-ubiquitination.

To further validate that BMI1-mediated chemoresistance took place through suppression of *ABCB1* by miR-3682-3p, we blocked *ABCB1* expression in T24/DDP&GEM cells by transfecting the cells with miR-3682-3p mimics or P-GP inhibitor, tariquidar (XR-9576). As shown in Figure 5G–5I, inhibition of *ABCB1* via transfecting with miR-3682-3p mimics or by its inhibitor XR-9576 significantly decreased viability (Figure 5G), promoted apoptosis (Figure 5H) and decreased dye efflux (Figure 5I) in BMI1-transduced cells. These experiments showed that miR-3682-3p could directly inhibit *ABCB1* gene through targeting its 3'-UTR, contributing to BMI1-mediated chemoresistance of bladder cancer cells.

### Clinical relevance among BMI1/miR-3682-3p/P-GP axis in bladder cancer specimens

**Figure 6:**
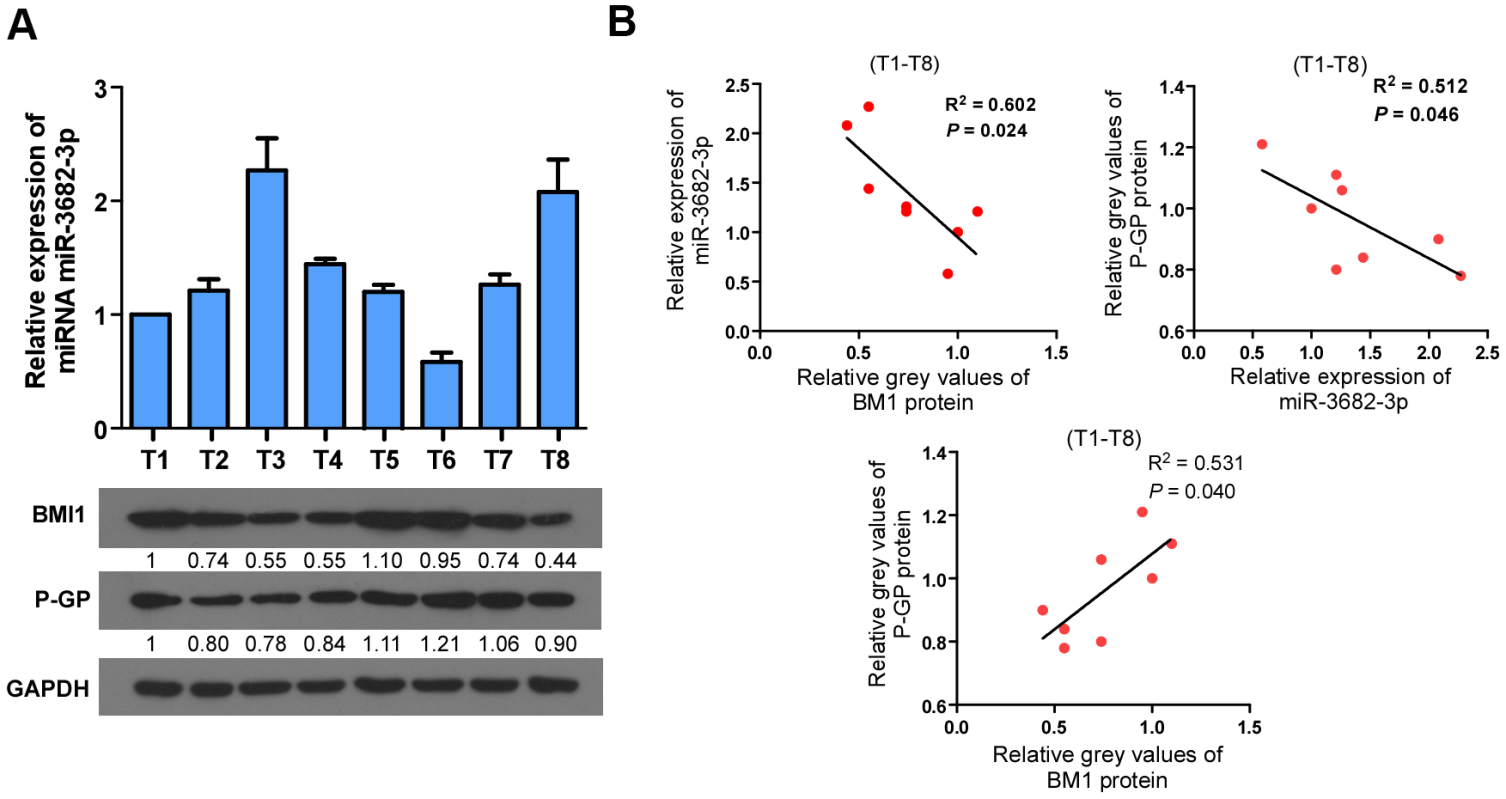
**Clinical relevance among BMI1/miR-3682-3p/P-GP axis in human bladder cancer.** (**A**) qRT-PCR or Western Blot detection of BMI1, P-GP and miR-3682-3p expression in 8 freshly collected human bladder cancer samples. (**B**) Correlation analyses among BMI1/miR-3682-3p/P-GP in these 8 bladder cancer tissues. P-GP: P-glycoprotein.

Next, we analyzed whether the BMI1/miR-3682-3p/P-GP axis found in bladder cancer cells were relevant in clinical. Eight fresh bladder cancer tissue specimens were collected and detected by q-RT-PCR and western blot (Figure 6A). BMI1 protein level correlated negatively with miR-3682-3p level (*P* = 0.024, R^2^ = 0.602), and correlated positively with P-GP protein level (*P* = 0.040, R^2^ = 0.531), while miR-3682-3p expression correlated negatively with P-GP protein level (*P* = 0.046, R^2^ = 0.512, Figure 6B). These results indicated that up-regulation of BMI1 in bladder cancer contributed to miR-3682-3p reduction, which in turn increased P-GP expression to enhance the chemoresistance of bladder cancer.

### Aberrant *BMI1* amplification contributes to BMI1 overexpression and chemoresistance in bladder cancer, which confers poor prognosis

**Figure 7:**
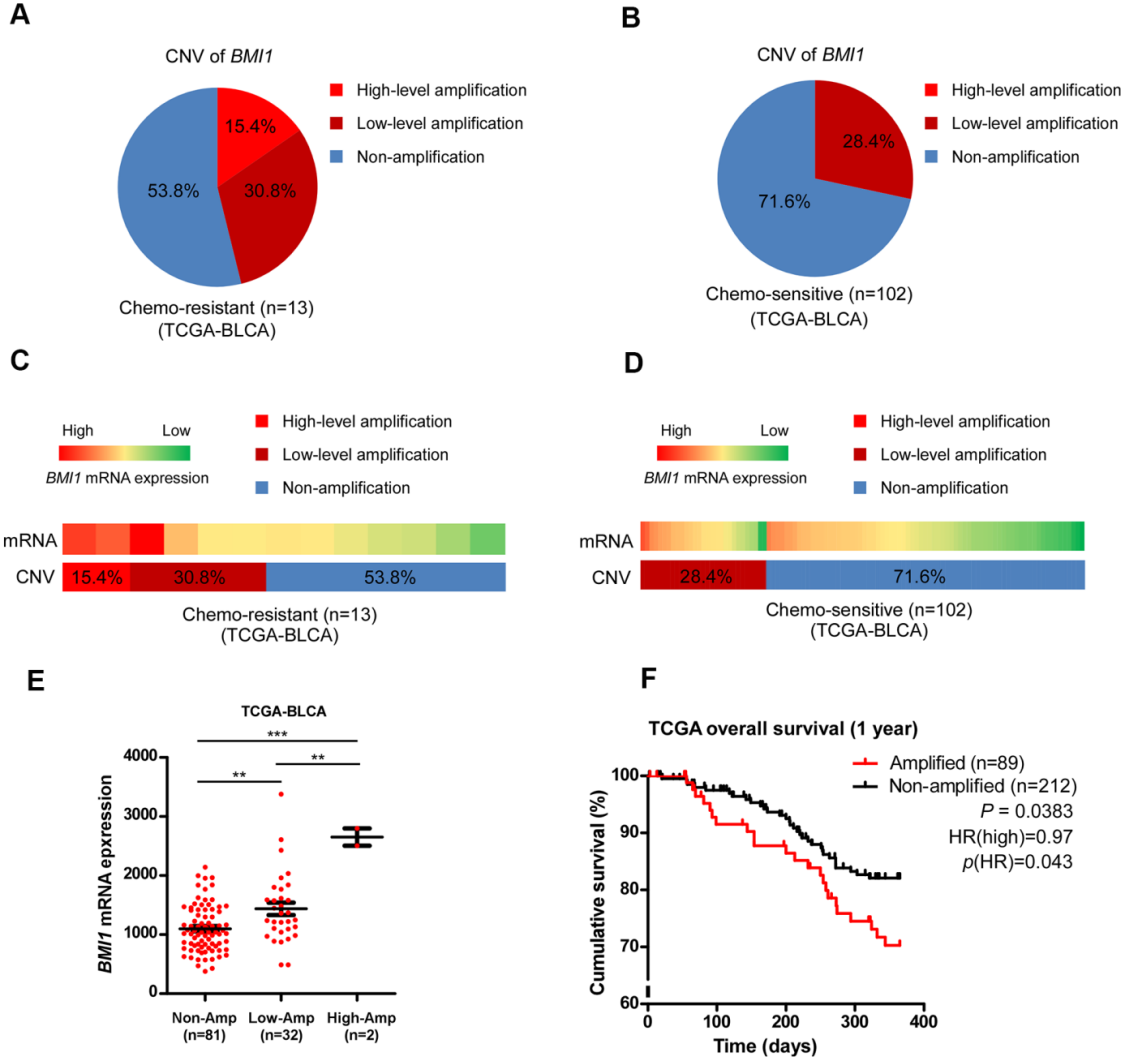
**Aberrant *BMI1* amplification contributed to BMI1 overexpression and chemoresistance in bladder cancer.** (**A**, **B**) Analysis of *BMI1* copy number variant (CNV) in bladder cancer patients resistant to chemotherapy (**A**) and that sensitive to chemotherapy (**B**) in TCGA-BLCA data sets. (**C**, **D**) *BMI1* gene CNV and corresponding mRNA expression in bladder cancer patients resistant chemotherapy (**C**) vs. that sensitive to chemotherapy (**D**) in TCGA-BLCA data sets (*P* = 0.0254). (**E**) *BMI1* gene CNV and corresponding mRNA expression in a TCGA bladder cancer data set (*P* < 0.001). (**F**) Kaplan-Meier analysis of overall survival for patients with amplified or non-amplified *BMI1* expression (*P* = 0.0383). *P < 0.05. **P < 0.01. ***P < 0.001. CNV: copy number variation; TCGA: The Cancer Genome Atlas; BLCA: Bladder Urothelial Carcinoma.

The *BMI1* locus is located on chromosome 10p12.2, that is generally amplified in multiple cancers [23, 48–50]. Compared with the *BMI1* copy number variation (CNV) in bladder cancer patients sensitive to chemotherapy in TCGA data sets, *BMI1* locus was highly amplified in bladder cancer patients resistant to chemotherapy (*P* = 0.0254, Figure 7A–7D), suggesting that ectopic *BMI1* amplification involved in the chemoresistance of bladder cancer. Consistently, *BMI1* mRNA level was significantly related to *BMI1* CNV (Figure 7C–7E). CNV in BMI1 expression correlated with neither p53 gene CNV nor p53 mRNA expression (Supplementary Figure 1). Importantly, bladder cancer with *BMI1* amplification predicted a worse survival than those without *BMI1* amplification (*P* = 0.0383; Figure 7F). Collectively, aberrant *BMI1* amplification contributes to BMI1 overexpression and chemoresistance in bladder cancer, which confers poor prognosis.

## DISCUSSION

This research mainly revealed that upon interaction with p53, BMI1 epigenetically repressed transcription of *miR-3682-3p*, which in turn increased P-GP expression, resulting in the enhanced drug efflux and chemoresistance of bladder cancer cells. BMI1 was detected to be over-expressed in bladder cancer specimens from GC-chemoresistant patients, which was consistent with the elevated BMI1 expression in established GC-resistant T24/DDP&GEM cells. BMI1 up-regulation, which could be attributed to the amplification of gene loci on the 10p12.2 chromosome, was related to the relapse and progression of bladder cancer. Herein, this study uncovered a novel mechanism underlying BMI1 up-regulation and BMI1-mediated activation of P-GP in GC-chemoresistant bladder cancer, and represented this protein as a potential target for GC-chemoresistant patients with bladder cancer.

BMI1 is a core component of PRC1 complex, which could function as an E3 ubiquitin ligase that transfers the mono-ubiquitination mark to the C-terminal tail of histone H2A at K118/K119 [51–54]. The complex is required to maintain the transcriptionally repressive state of many genes through catalyzing the H2AK119ub1, an epigenetic marker related to gene suppression [45–47, 51]. Ectopic H2AK119ub1 expression has been correlated with the poor prognosis of some tumors [55]. BMI1 enhanced the enzymatic activities of RING1B to mono-ubiquitinate H2AK119 and repress gene transcription [51]. Knockout of Bmi-1 results in a decrease in H2AK119ub1 [56] concomitant with derepression of Hox gene silencing [45, 57]. BMI1 is frequently up-regulated in a variety of cancer, and its over-expression is associated with poor prognosis [19, 58]. Multiple researches have shown that BMI1 is involved in chemoresistance, while inhibiting BMI1 can make cancer cells sensitive to chemotherapy [27, 28, 59]. Consistently, our study revealed that BMI1 was overexpressed in GC-chemoresistant bladder cancer tissues, which confers poor prognosis. Overexpressing BMI1 significantly enhanced, whereas silencing BMI1 attenuated drug efflux and chemoresistance of bladder cancer cells. Mechanically, upon interaction with p53, BMI1 overexpression increased its occupancy on *miR-3682-3p* gene promoter concomitant with an increase in H2AK119ub1, leading to the reduced transcription of *miR-3682-3p* gene followed by derepression of *ABCB1* gene.

*ABCB1* is a member of the superfamily of ATP-binding cassette transporters. As an important MDR related gene, *ABCB1* gene encodes P-GP protein and participates in intracellular drug efflux, leading to decreased drug accumulation and development of resistance to anticancer drugs [60–64]. Ectopic P-GP level was inversely related to the prognosis in bladder cancer [64]. Herein, our study identified a novel microRNA post-translationally regulating P-GP overexpression in GC-chemoresistant patients with bladder cancer. Suppression of miR-3682-3p by BMI1 restored P-GP expression in bladder cancer, resulting in the chemoresistant to GC, and tumor relapse and progression.

Currently, most of the chemotherapies for bladder cancer are platinum-based combined regimens in clinic, owing to the low response rate of single drug therapy [65–67]. As the first-line treatment, combined chemotherapy of cisplatin and gemcitabine is the most commonly used for advanced MIBC patients, but its response rate is still not more than 50% [3]. In this study, we established T24/DDP&GEM cells resistant to cisplatin and gemcitabine, and investigated the regulation role of BMI1/miR-3682-3p/P-GP axis in T24/DDP&GEM cells and in bladder cancer tissues of patients resistant to GC chemotherapy. Suppression of P-GP by miR-3682-3p mimics or XR-9576 could significantly reverse BMI1-mediated chemoresistance of bladder cancer cells, presenting miR-3682-3p or XR-9576 as a potential adjuvant agent in GC-chemoresistant bladder cancer with ectopic BMI1 expression. XR-9576, also named tariquidar, is the 3^rd^ generation of P-GP inhibitors. XR-9576 has high affinity with P-GP, can bind to P-GP non-competitively and strongly suppresses its activity [68]. XR-9576 did not interfere with the pharmacokinetics of doxorubicin, paclitaxel, or vinorelbine in chemotherapy of patients with solid tumours [69, 70]. Several clinical trials using XR-9576 in combination with chemotherapy revealed that XR-9576 is a potent P-GP antagonist without significant side effects [69–72], representing XR-9576 as a promising treatment for MDR bladder cancer with ectopic BMI1 expression.

This study demonstrated that the elevated BMI1 expression observed in GC-resistant patients with bladder cancer could be due to genomic amplification of 10p12.2, which conferred poor prognosis. BMI1 overexpression dramatically promoted drug efflux, enhanced viability and decreased apoptosis of bladder cancer cells upon chemotherapy with DDP or GEM, whereas BMI1 downregulation reversed this effect. Mechanically, *miR-3682-3p* was identified as a downstream target of p53. Upon interaction with p53, BMI1 could repress the transcription of *miR-3682-3p* gene following an increase in H2AK119ub1, leading to derepression of *ABCB1* gene. Moreover, suppression of P-GP by miR-3682-3p mimics or XR-9576 could significantly reverse BMI1-mediated chemoresistance of bladder cancer cells. The results provided a novel insight into the portion of mechanism underlying BMI1-mediated chemoresistance in bladder cancer, presenting BMI1 as a valuable prognosis indicator and potential therapeutic target for GC-resistant bladder cancer.

## Supplementary Material

Supplementary Figure 1
