# Supplementary material for: BMI1 activates P-glycoprotein via transcription repression of miR-3682-3p and enhances chemoresistance of bladder cancer cell
Source: Aging (Albany NY). 2021 Jul 16;13(14):18310–30. doi: 10.18632/aging.203277 (PMC8351696; doi:10.18632/aging.203277)
Supplement: Supplementary Figure 1 [file aging-13-203277-s001.pdf]

SUPPLEMENTARY FIGURE

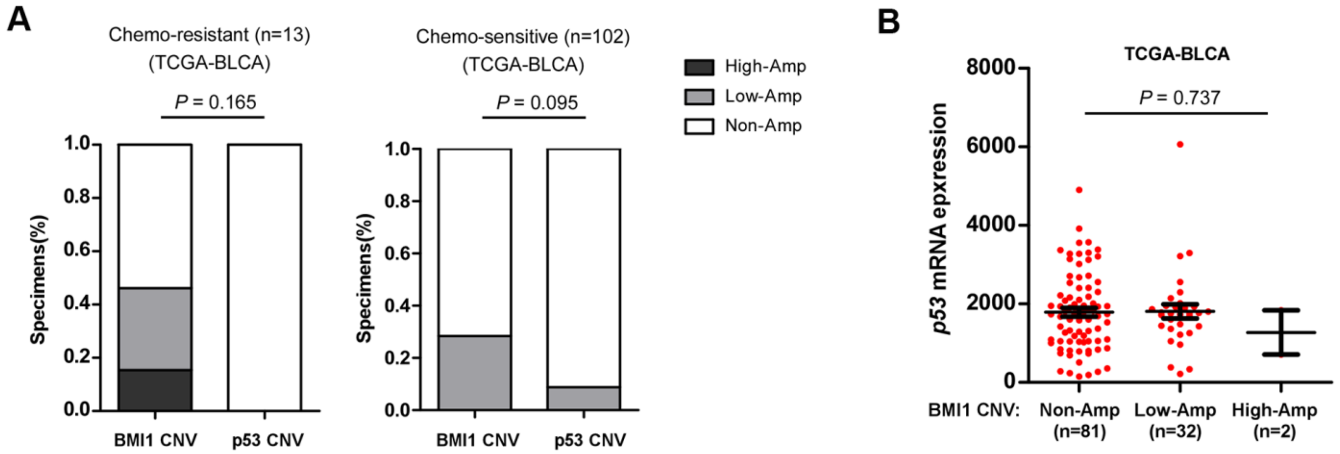

Supplementary Figure 1. (**A**) Correlation analyses of CNV in BMI1 expression with p53 gene CNV in chemo-resistant (left) and chemo-sensitive (right) bladder cancer specimens from TCGA dataset. (**B**) BMI1 gene CNV and p53 mRNA expression in a TCGA-BLCA data set (*P* = 0.737). CNV: copy number variant; TCGA: The Cancer Genome Atlas; BLCA: Bladder Urothelial Carcinoma.
